# Chromatin structure predicts survival in glioma patients

**DOI:** 10.1038/s41598-022-11019-9

**Published:** 2022-05-17

**Authors:** Matthew C. Garrett, Rebecca Albano, Troy Carnwath, Sanjit Shah, Daniel Woo, Michael Lamba, David R. Plas, Aditi Paranjpe, Krishna Roskin, Chuntao Zhao, Richard Lu

**Affiliations:** 1 Department of Neurosurgery, University of Cincinnati College of Medicine, Cincinnati, OH 45267 USA; 2 University of Cincinnati College of Medicine, Cincinnati, OH 45267 USA; 3 Department of Neurology, University of Cincinnati College of Medicine, Cincinnati, OH 45267 USA; 4 Department of Radiation Oncology, University of Cincinnati, Cincinnati, OH 45267 USA; 5 Department of Cancer Biology, University of Cincinnati, Cincinnati, OH 45267 USA; 6 Division of Biomedical Informatics, Bioinformatics Collaborative Services, Cincinnati Children’s Hospital Medical Center, Cincinnati, OH USA; 7 Department of Cancer Biology, Cincinnati Children’s Hospital, Cincinnati, OH 45267 USA

**Keywords:** Cancer, Cell biology, Computational biology and bioinformatics, Genetics

## Abstract

The pathological changes in epigenetics and gene regulation that accompany the progression of low-grade to high-grade gliomas are under-studied. The authors use a large set of paired atac-seq and RNA-seq data from surgically resected glioma specimens to infer gene regulatory relationships in glioma. Thirty-eight glioma patient samples underwent atac-seq sequencing and 16 samples underwent additional RNA-seq analysis. Using an atac-seq/RNA-seq correlation matrix, atac-seq peaks were paired with genes based on high correlation values (|r^2^| > 0.6). Samples clustered by IDH1 status but not by grade. Surprisingly there was a trend for IDH1 mutant samples to have more peaks. The majority of peaks are positively correlated with survival and positively correlated with gene expression. Constructing a model of the top six atac-seq peaks created a highly accurate survival prediction model (r^2^ = 0.68). Four of these peaks were still significant after controlling for age, grade, pathology, IDH1 status and gender. Grade II, III, and IV (primary) samples have similar transcription factors and gene modules. However, grade IV (recurrent) samples have strikingly few peaks. Patient-derived glioma cultures showed decreased peak counts following radiation indicating that this may be radiation-induced. This study supports the notion that IDH1 mutant and IDH1 wildtype gliomas have different epigenetic landscapes and that accessible chromatin sites mapped by atac-seq peaks tend to be positively correlated with expression. The data in this study leads to a new model of treatment response wherein glioma cells respond to radiation therapy by closing open regions of DNA.

## Introduction

Gliomas are the most common primary brain tumor and result in over 15,000 deaths a year in the United States alone^1^. These tumors are often initially discovered as slow-growing low-grade gliomas (Grade II gliomas)^2,3^. However, inevitably these tumors undergo a transformation into faster growing more aggressive higher-grade tumors (Grade III and Grade IV gliomas). Following diagnosis, standard clinical management includes maximal safe resection and adjuvant treatment (chemotherapy and radiation)^1,3–5^. This often leads to a period of clinical stability where there is no visible tumor on serial MRI scans. However, the tumors inevitably recur. While primary grade IV gliomas (i.e. glioblastomas) can have periods of stability following resection and adjuvant therapy, once they recur, recurrent glioblastomas are typically treatment resistant and clinical stability is rare^6,7^. Whether this is due to a natural progression of the tumor or as a response to typically used DNA-damaging therapies (radiation and chemotherapy) is being investigated^8^.

The transformation from a normal cell to a low-grade glioma and eventually a high-grade glioma involves the dysregulation of many gene pathways and cellular processes^9,10^. Much attention has been paid to deletions (e.g. *PTEN*^11,12^, *p53*^13^, 1p/19q^14,15^) and amplifications (*EGFR*^16^, *MYC*^17^) of specific gene regions that are frequently encountered in these tumors. However, there is a similar and equally important pathology in the epigenetic dysregulation of these gene pathways that has received less attention. A thorough exploration of the epigenetic landscape of these tumors may lead to the discovery of additional important biomarkers.

The discovery of the IDH1 mutation (frequently found in low-grade gliomas and secondary high-grade gliomas) has brought additional focus and attention to the problem of epigenetic dysregulation in gliomas. The presumed mechanism of the IDH1 mutation is to methylate cytosine nucleotides in DNA and induce a more closed inaccessible DNA chromatin with subsequent gene down-regulation^18–20^ however atac-seq data on IDH1 mutant glioma samples is limited.

Atac-seq is a relatively new sequencing technology that identifies open accessible DNA regions (e.g. “peaks”) with very little cellular input enabling the analysis of surgically resected samples. Its low-cost and relative ease has led to wide-spread use across multiple cell types. However, when atac-seq data is used in isolation without additional lines of supporting evidence, it has been difficult to determine how best to interpret the biological significance of these open regions (measured as “peaks” from aligned sequencing reads). Data sets involving multiple modalities (atac-seq and RNA-seq) on the same samples would be useful in supporting or refuting these assumptions. To address these short-comings this study combines a large set of atac-seq and RNA-seq data from surgically removed glioma specimens to create an atac-seq/RNA-seq matrix to identify highly correlated peaks and genes. By correlating these data sets with demographic and survival data we have identified open DNA regions that have prognostic and thereby likely biological significance.

## Methods

### Generation of clinical database

The University of Cincinnati maintains an IRB-approved biorepository of surgically resected specimens with associated clinical and demographic information including medical record number, diagnosis, name, gender, age, date of collection and survival. A collection of 38 specimens were chosen from the bio-repository with a goal to obtain a heterogenous mixture of low-grade and high-grade specimens as well as those with long and short survival. Specimens were between 2 and 9 years old prior to selection to allow for adequate follow-up for survival analysis. All specimens denoted as “Grade II,” “Grade III,” or “Grade IV primary” are taken from the patient’s initial surgery and have not been exposed to any chemotherapy or radiation treatment. All grades were assigned based on the WHO guidelines of that time. Since then, grading guidelines have changed to include more molecular markers (e.g. IDH1, p53, 1p/19q)^21^. Following the initial surgery all patients underwent standard therapy including temozolomide and radiation. Survival was calculated as time between surgical resection and death. The presence of the IDH1 mutation was determined via either immunostaining or by direct sequencing.

### Protocol for generating Atac-seq libraries

Nuclei were isolated from patient tumor tissue as described by Habib et al.^22^, with slight modifications. Briefly, frozen tissue samples (< 0.5 cm) were incubated on ice in 1 mL of Nuclei EZ Lysis Buffer (Sigma, #Nuc-101). The tissue was then homogenized using a glass tissue grinder. 1 mL of additional EZ Lysis Buffer was added, the tissue was incubated on ice for 5 min, and then filtered through a 70 μm cell strainer. Nuclei were collected by centrifugation and resuspended in 1 mL ATAC-resuspension buffer (10 mM Tris–HCl (pH 7.4), 10 mM NaCl, 3 mM MgCl_2_, and 0.1% Tween-20) and filtered through a 40 μm cell strainer. 1 mL of ATAC-resuspension buffer was then added and the nuclei were filtered through a 5 μm cell strainer. ATAC-seq library preparation was performed as previously described^23^.

### RNA-seq library creation

Frozen patient tumor tissue (< 0.5 cm) was placed into pre-chilled RNA-ice later (Invitrogen, #AM7030) for at least 24 h at − 20 °C. The tissue was then homogenized with Lysis/Binding Buffer (Invitrogen, #00671513) in lysing matrix D tubes (MP, #116913050-CF) for 40 s at 6 m/s, using the MP FastPrep-24 5G Instrument (MP, #116005500). RNA (≥ 200 nucleotides) was then purified using the Quick-RNA MiniPrep Plus Kit (Zymo Research, #R1057).

### Processing of ATAC-seq data

ATAC-seq reads in FASTQ format were first subjected to quality control to assess the need for trimming of adapter sequences or bad quality segments. The programs used in these steps were FastQC v0.11.7, Trim Galore! v0.4.2 and cutadapt v1.9.1. The trimmed reads were aligned to the reference human genome version GRCh38/hg38 with the program HISAT2 v2.0.5. Aligned reads were stripped of duplicate reads with the program sambamba v0.6.8. Peaks were called using the program MACS v2.1.2 using the broad peaks mode.

To obtain the consensus set of unique peaks, called peaks from all samples are merged at 50% overlap using BEDtools v2.27.0. The consensus peaks, originally in BED format were converted to a Gene Transfer Format (GTF) to enable fast counting of reads under the peaks with the program featureCounts v1.6.2. Each feature in the GTF file has the value “peak” on the second column. Peaks located on chromosomes X, Y and mitochondrial DNA are excluded from further analysis. Raw read counts are normalized with respect to library size and transformed to log2 scale using rlog() function in R package DESeq2 v1.26.0.

### Random forest regression model

Log-transformed data were filtered to remove low variance peaks using the VarianceThreshold function from scikit-learn v0.24.1 using a threshold of 0.45. The resulting 8439 features were used to build a random forest regression model using RandomForestRegressor from the ensemble module of scikit-learn. The options used was n_estimators: 1000, max_samples = 0.9, oob_score = True. Permutation importance was used to rank the peaks. The out-of-bag R^2^ value for the regression was 0.31.

### Elastic-net regularized generalized linear models

We selected the top 20 peaks with highest mean feature importance value from 8439 peaks. To assess the survival predictive power of clinical metadata and top 20 ATAC-seq peaks, we trained elastic-net regularized generalized linear models using ‘glmnet’ package in R. We tested the performance of three different models that are built using following features (1) top 20 ATAC-seq peaks (2) clinical metadata only; (3) clinical metadata + top 20 ATAC-seq peaks. Leave one out cross validation imputation was applied and alpha parameter was explored between 0 and 1 with incrementing step size of 0.1. Out of 36 samples, IDH mutation information of 4 samples was not available. These 4 samples were excluded from models which include clinical metadata as features. Prediction accuracy of models was assessed by computing R^2^ value.

### Processing of RNA-seq data

The quality control check on RNA-seq reads was performed with FastQC v0.11.7. Adapter sequences and bad quality segments were trimmed using Trim Galore! v0.4.2 and cutadapt v1.9.1. The trimmed reads were aligned to the reference human genome version GRCh38/hg38 with the program STAR v2.6.1e. Duplicate aligned reads were removed using the program sambamba v0.6.8. Gene-level expression was assessed by counting features for each gene, as defined in the NCBI’s RefSeq database. Read counting was done with the program featureCounts v1.6.2 from the Rsubread package. Raw counts were normalized with respect to library size and transformed to log2 scale using rlog() function in R package DESeq2 v1.26.0.

### ATAC-seq and RNA-seq correlation analysis

Sixteen samples have atac-seq chromatin accessibility as well as RNA-seq gene expression data which is used for correlation analysis. To compare the chromatin accessibility and gene expression, we identified the peak-gene pairs which are located within same topologically associating domain (TAD). Peaks having at least 90% overlap with TAD are selected for further analysis. Out of 8439 peaks, 8419 peaks show ≥ 90% overlap with TAD region and 8416 peaks have at least 1 gene within the overlapping TAD. Pearson correlation coefficient was calculated for each peak-gene pair.

### Analysis of differential chromatin accessibility

The significant changes of chromatin accessibility in each subgroup (Grade II, Grade III, Grade IV Primary and Grade IV Recurrent) were assessed with the R package DESeq2 v1.26.0. The 8439 most important peaks from the random forest regression model are selected for the differential analysis. Samples from a specific subgroup (e.g. Grade II) are compared against remaining samples from the dataset. Differential peaks with log_2_FoldChange ≥ 0.3 and FDR ≤ 0.05 are selected for motif enrichment analysis using HOMER v4.10. Remaining peaks from initial set of 8439 peaks are used as background data set in HOMER analysis. Only known human motifs from CIS-BP 2.0 are considered.

### Radiation treatment

Three previously characterized patient-derived cultures (HK296^24^, HK357^24^, TS603^25^) underwent a radiation dose of 9 Gy in three fractions in a XenX (X-Strahl, Suwanee, GA) pre-clinical cabinet irradiator, with output calibrated using NIST-traceable instruments. The instrument parameters were: 220 kVp, 0.67 mm Cu HVL, dose rate ~ 6 cGy/s, delivered using a 100 mm × 100 mm collimator with 2 cm backscatter below the cell plates.

### Sequencing database

The human database is publicly available from GEO (https://www.ncbi.nlm.nih.gov/geo/query/acc.cgi?acc=GSE295378). All experimental protocols were approved by the University of Cincinnati IRB committee. This study was used de-identified patient specimens and was designated “Not human research” 19-02-25-02 (5/3/2019). Consent is not applicable.

### Human database

The creation of the human database followed all methods in accordance with relevant guidelines and regulations. This study was used de-identified patient specimens and was designated “Not human research” 19-02-25-02 (5/3/2019). Consent is not applicable.

### Consent to participate

This study does not involve human subjects.

## Results

### Atac-seq/RNA-seq correlation matrix creates chromatin maps with candidate target genes

**Figure 1:**
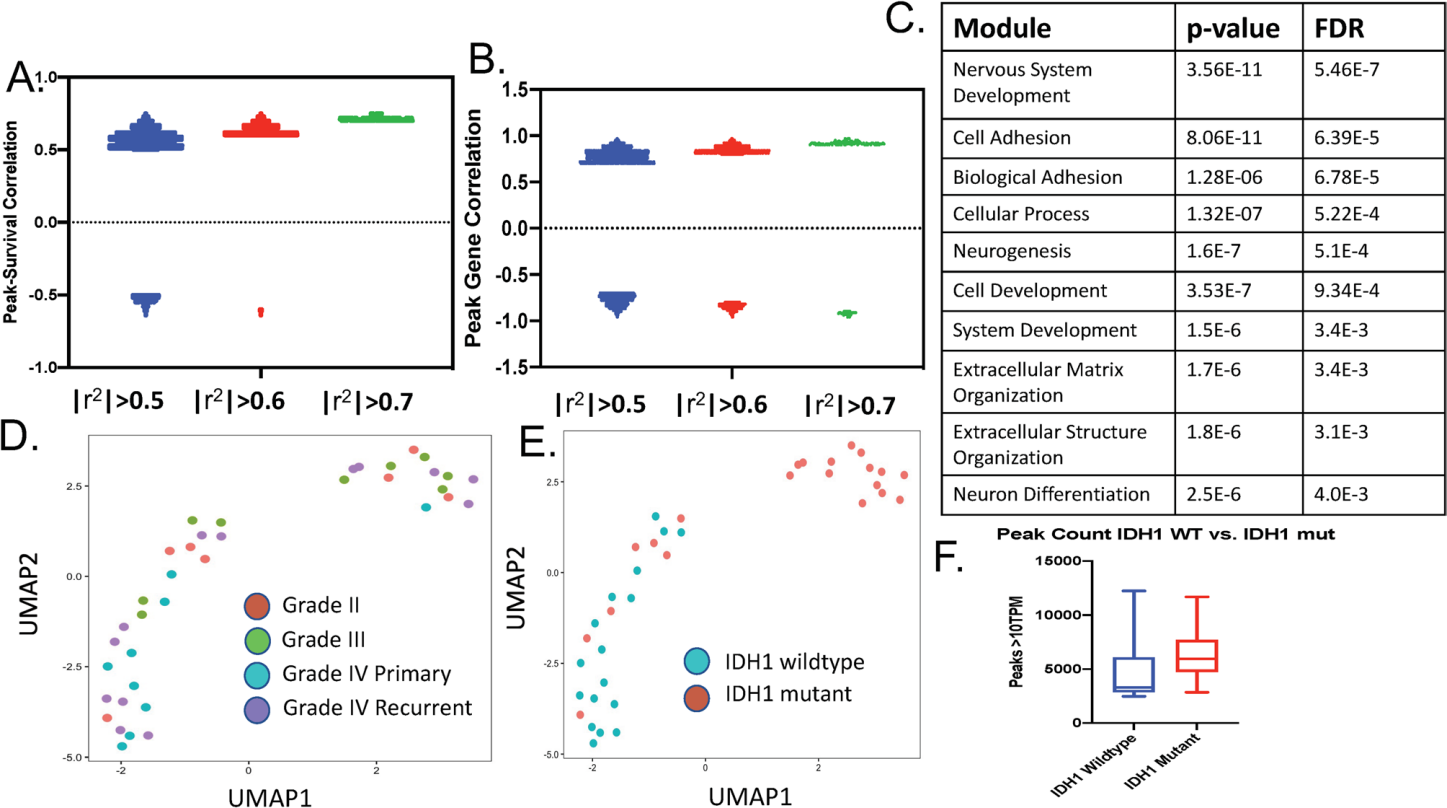
(**A**) Univariate correlations between peaks and survival are plotted above for |r^2^| > 0.5, |r^2^| > 0.6, and |r^2^| > 0.7. The vast majority of the correlations between peaks and survival are positively correlated. (**B**) Univariate correlations between peaks and genes are plotted above for |r^2^| > 0.5, |r^2^| > 0.6, and |r^2^| > 0.7. The vast majority of the correlations between peaks and genes are positively correlated. (**C**) Peak-gene correlations of |r^2^| > 0.6 were subjected to gene ontology analysis. Significantly enriched gene modules are shown above. (**D,E**) UMAP clustering of all 38 samples coded by grade (**D**) and IDH1 status (**E**). (**F**) Total peaks (Transcript per million).

This study includes thirty-eight glioma specimens spanning all grades (Grade II-7 specimens, Grade III-9 specimens, Grade IV primary-9 specimens, Grade IV recurrent-13 specimens). Seventeen specimens were determined to have an IDH1 mutation, 17 specimens were determined to be IDH1 wildtype. In four specimens, the IDH1 mutant status could not be determined. The collection includes three paired specimens in which a single patient underwent two surgeries (MG 18/MG19, MG20/MG21, MG27/28). Sixteen samples were of sufficient quality to allow RNA-sequencing analysis. The demographic and clinical information is shown in Table 1. Restricting analysis to those peaks with a univariate correlation of |r^2^| ≥ 0.6 revealed that the majority of peaks were positively correlated with survival (Fig. 1A) and positively correlated with gene expression (Fig. 1B). Gene ontology enrichment analysis of the identified correlated genes (peak-survival correlation |r^2^| ≥ 0.6 and peak-gene correlation |r^2^| ≥ 0.6) identified “Nervous System Development” as the most enriched gene module (Fig. 1C). Clustering using Uniform Manifold Approximation and Projection (UMAP) identified two clusters that segregated by IDH1 mutant status (Fig. 1D,E). While it is widely presumed that the IDH1 mutation induces DNA methylation and a closed conformation^18–20^, there was a non-significant trend (p = 0.1) towards IDH1 mutant samples having more peaks (Fig. 1F).

**Table 1:** Demographic and clinical information for 38 glioma specimens spanning all grades (Grade II-7 specimens, Grade III-9 specimens, Grade IV prary-9 specimens, Grade IV recurrent-13 specimens).

Sample number	Age	Gender	Pathology	Grade	Primary/recurrent	IDH1	Survival (months)	RNA-seq
MG1	63	Female	Glioblastoma	IV	Recurrent	WT	5	Yes
MG2	77	Male	Glioblastoma	IV	Recurrent	WT	9	Yes
MG3	67	Female	Glioblastoma	IV	Primary	WT	51	Yes
MG4	30	Female	Anaplastic astrocytoma	III	Primary	WT	44	Yes
MG5	69	Male	Glioblastoma	IV	Primary	WT	3.5	Yes
MG6	49	Male	Glioblastoma	IV	Recurrent	WT	5.5	Yes
MG7	63	Male	Oligodendroglioma	II	Primary	Mut	69	Yes
MG8	76	Male	Glioblastoma	IV	Primary	WT	14	No
MG9	25	Male	Anaplastic oligodendroglioma	III	Primary	Mut	66	Yes
MG10	38	Female	Oligodendroglioma	II	Primary	Mut	65	Yes
MG11	46	Male	Anaplastic oligodendroglioma	III	Primary	Mut	64	Yes
MG12	54	Female	Glioblastoma	IV	Recurrent	WT	8	Yes
MG13	30	Female	Anaplastic oligodendroglioma	III	Primary	Mut	50	Yes
MG14	63	Female	Anaplastic oligodendroglioma	III	Primary	Mut	25	Yes
MG15	49	Female	Glioblastoma	IV	Primary	WT	4	Yes
MG16	48	Male	Oligodendroglioma	II	Primary	Mut	21	Yes
MG17	33	Female	Oligodendroglioma	II	Primary	Mut	14	Yes
MG18	40	Female	Anaplastic astrocytoma	III	Primary	–	34	No
MG19	43	Female	Glioblastoma	IV	Recurrent	–	4	No
MG20	44	Female	Glioblastoma	IV	Primary	Mut	53	No
MG21	48	Female	Glioblastoma	IV	Recurrent	Mut	7	No
MG22	64	Male	Glioblastoma	IV	Primary	WT	5	No
MG23	32	Female	Glioblastoma	IV	Recurrent	–	41	No
MG24	55	Male	Anaplastic oligodendroglioma	III	Primary	Mut	97	No
MG25	47	Male	Astrocytoma	II	Primary	Mut	88	No
MG26	31	Male	Anaplastic astrocytoma	III	Primary	Mut	89	No
MG27	58	Female	Glioblastoma	IV	Primary	WT	16	No
MG28	58	Female	Glioblastoma	IV	Recurrent	WT	13	No
MG29	63	Male	Glioblastoma	IV	Recurrent	–	4	No
MG30	49	Male	Glioblastoma	IV	Recurrent	WT	14	No
MG31	55	Male	Oligodendroglioma	II	Primary	Mut	58	No
MG32	32	Male	Astrocytoma	II	Primary	Mut	57	No
MG33	49	Female	Glioblastoma	IV	Primary	WT	16	No
MG34	21	Female	Glioblastoma	IV	Recurrent	WT	5	No
MG35	50	Male	Glioblastoma	IV	Recurrent	Mut	5	No
MG36	69	Female	Glioblastoma	IV	Primary	WT	12	No
MG37	37	Female	Glioblastoma	IV	Recurrent	Mut	39	No
MG38	66	Male	Anaplastic oligodendroglioma	III	Primary	WT	18	No

Seventeen specimens were determined to have an IDH1 mutation, 17 specimens were determined to be IDH1 wildtype. In four specimens, the IDH1 mutant status could not be determined. The collection includes three paired specimens in which a single patient underwent two surgeries (MG 18/MG19, MG20/MG21, MG27/28). Sixteen samples were of sufficient quality to allow RNA-sequencing analysis.

### Prognostic model predicts identifies atac-seq peaks associated with grade/survival

**Figure 2:**
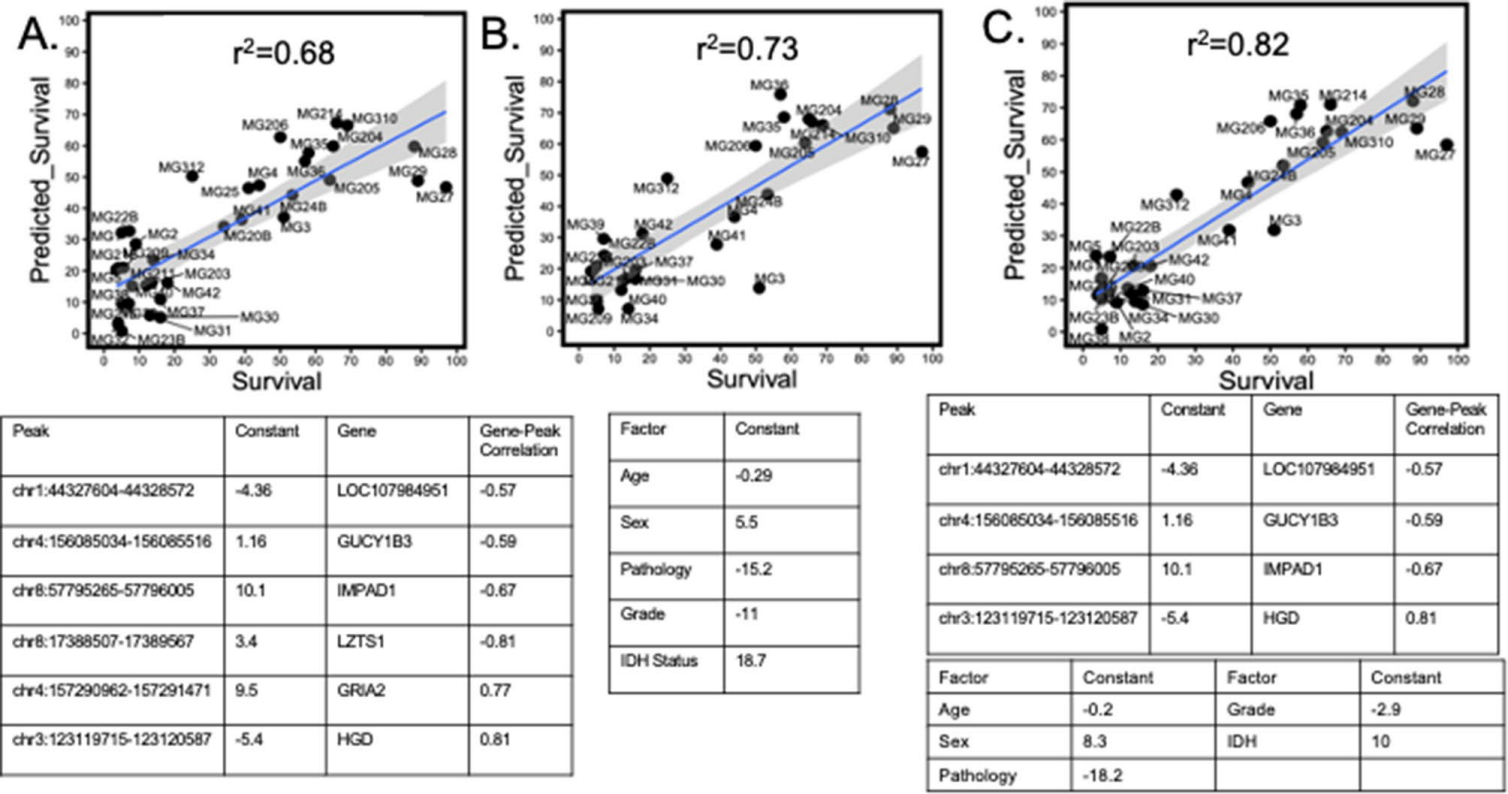
(**A**) The six most predictive atac-seq peaks were used to create a linear regression model to predict survival. The constants associated with this linear equation are shown in column two and the most correlated gene in column 3. (**B**) Six typically used clinical and demographic variables were used to create a linear regression model to predict survival. (**C**) Combining atac-seq peaks and clinical variables into a linear regression model yielded the most accurate prediction model.

Restricting the focus to only those genes with the most prognostic value, a random forest model identified 8439 peaks that were predictive of survival. The majority of these peaks mapped to intergenic regions. Using the atac-seq/RNA-seq correlation matrix, 29,679 genes were correlated with the identified 8439 peaks (gene-peak correlation |r^2^| ≥ 0.6). Contrary to the hypothesis that peaks target the “nearest gene”, only 1863 (2.5%) of these correlations were between a peak and its “nearest gene”. Linear regression models identified six peaks that predicted survival with a high degree of accuracy (R^2^ = 0.6845). The six most closely correlated target gene regions are shown in Fig. 2A. Four of the annotated genes have reported roles in cancer. *GUCY1B3*^26,27^ and *IMPAD1*^28,29^ are reported oncogenes while *GRIA2*^30^ and *LZTS1*^31^ are reported tumor suppressors. This peak linear regression model was compared against a model using established predictive clinical variables (age, gender, grade, pathology and IDH1 status) which yielded a higher degree of accuracy (R^2^ = 0.7271 Fig. 2B). Combining the atac-seq peaks and clinical variables created the most accurate model (R^2^ = 0.8164 Fig. 2C) indicating that four of the atac-seq peaks have predictive value independent of traditionally used clinical variables.

### Recurrent grade IV gliomas are associated with fewer open chromatin sites

**Figure 3:**
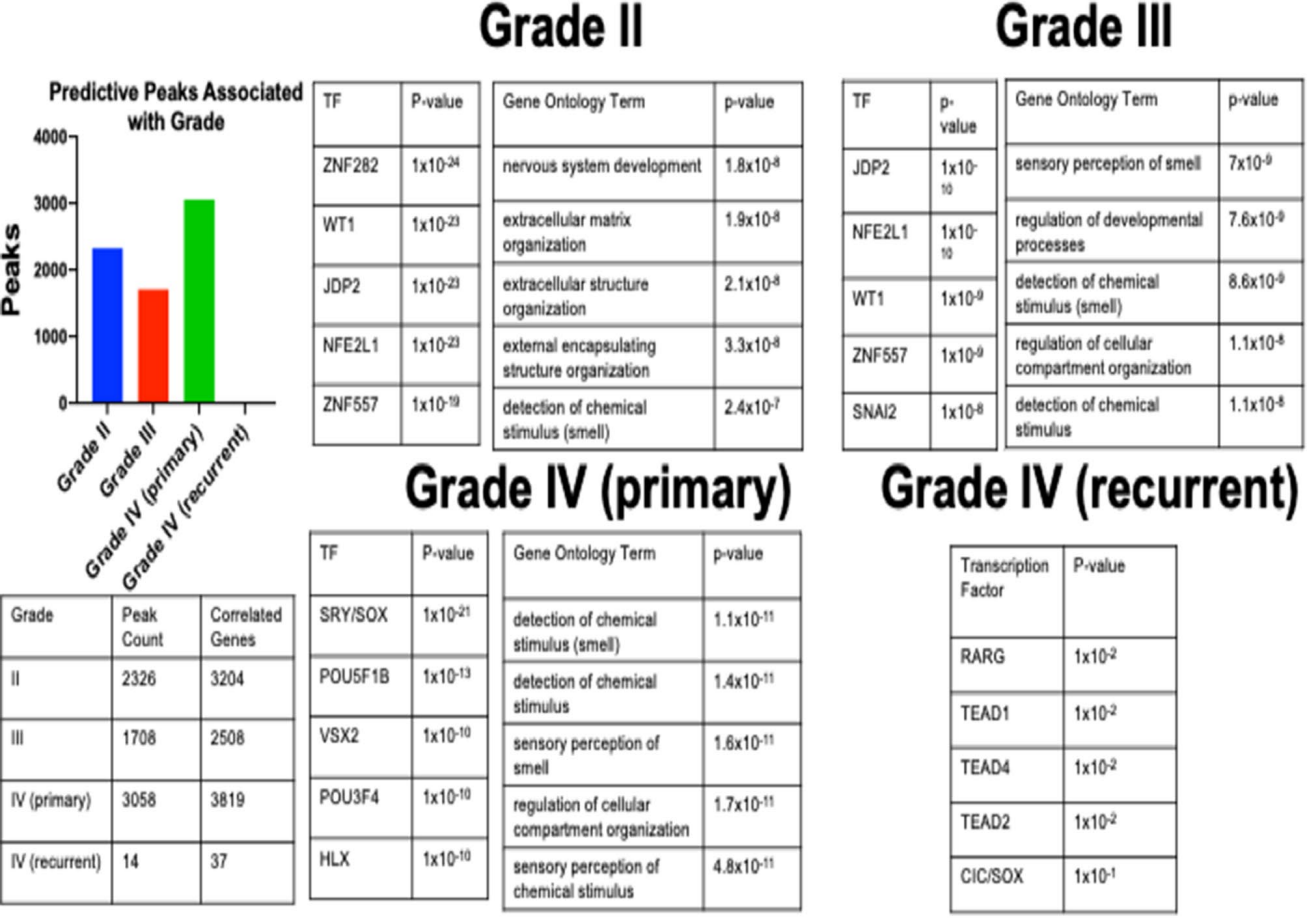
Each of the 8349 peaks was assigned to one of the four grades (Grade II, Grade III, Grade IV primary, Grade IV recurrent) based on representation in that group. These peaks underwent HOMER analysis to determine enriched transcription factor motifs and gene ontology based on the associated genes. Grade IV recurrent samples had very few peaks and no significantly enriched gene modules.

Each grade was compared to all others to identity any differentially represented atac-seq peaks (Fig. 3). These grade-specific peaks then underwent HOMER motif analysis to determine which likely transcription factors drove gene expression in each grade. Finally, peak-associated genes were identified and underwent gene ontology analysis to determine candidate target gene pathways for each grade. Grade II (2326 peaks, 3204 genes), grade III (1708 peaks, 2508 genes), and grade IV (primary) (3058 peaks, 3819 genes) samples had similar and heavily overlapping sets of transcription factor motifs and enriched gene modules (Fig. 3). In contrast, grade IV recurrent (14 peaks, 37 genes) had relatively few peaks and no significantly enriched gene modules.

**Figure 4:**
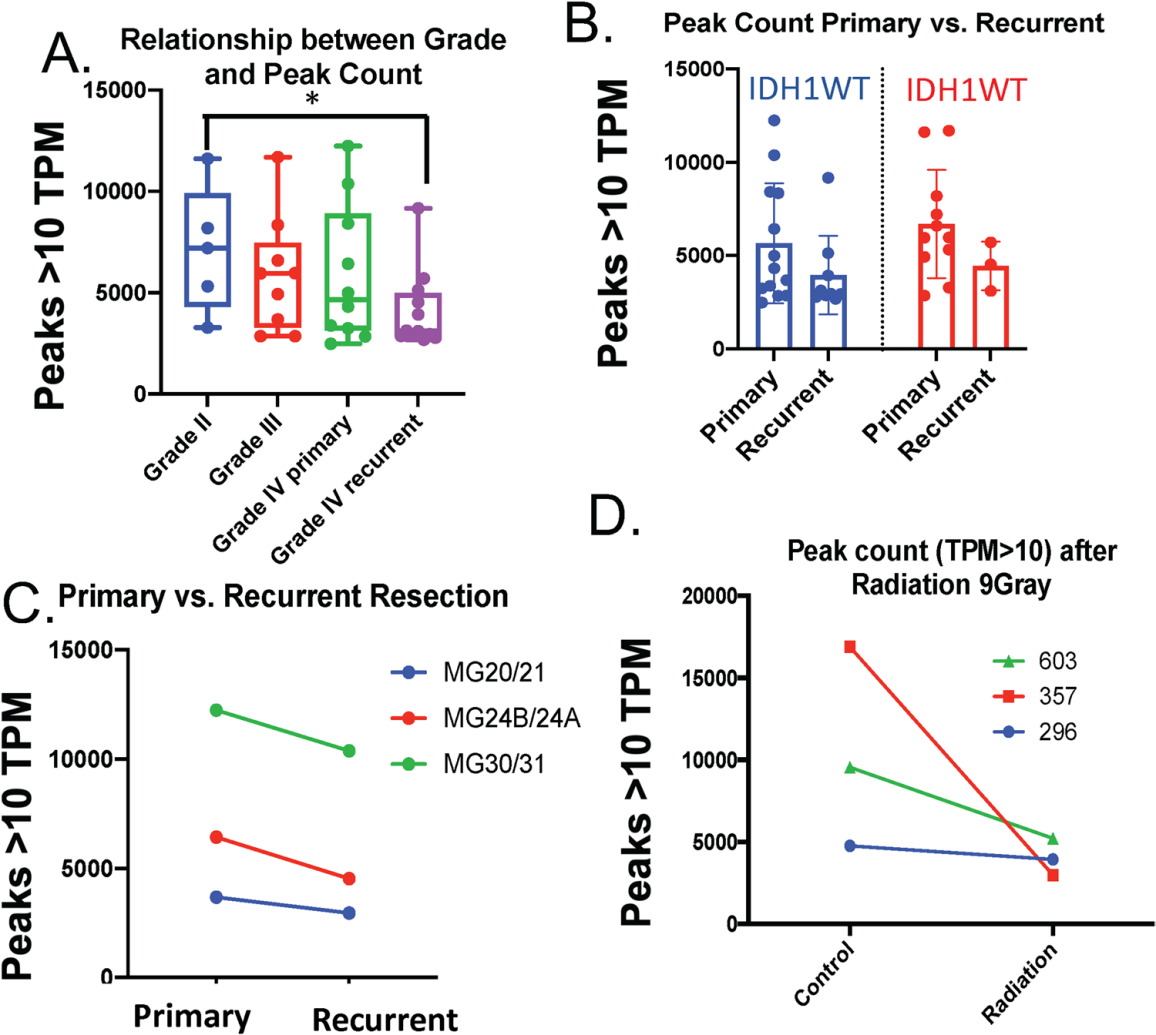
(**A**) The total peaks (transcripts per million > 10) were calculated for each sample and tabulated by grade. Grade II samples had significantly more peaks than Grade IV recurrent samples (ANOVA). (**B**) The total peak counts for three paired samples are shown. (**C**) Samples were divided by IDH1 status and by primary vs. recurrent status. (**D**) Three patient derived glioblastoma cell lines were subjected to atac-seq analysis before and after 9 grey radiation in three fractions.

To determine if this finding was specific to the identified 8439 peaks or part of a larger trend, the total peak count was calculated for each of the grades. Similar to the previous finding, grade IV (recurrent) samples had significantly fewer total peaks than grade II samples (p = 0.02) (Fig. 4A,B). This collection contains three “paired” samples each of which were grade IV that underwent resection followed by standard adjuvant treatment (temozolomide and radiation) and later a second resection. In each case, the later sample (“recurrent” sample) had fewer peaks (Fig. 4C). This invites two possibilities. The first possibility is the decrease in peak count is a selection process where only cells with fewer peaks grow back. The second possibility is that the decrease in peak count is a consequence of the clinical treatment (e.g. surgery, temozolomide and radiation). In support of the latter induction hypothesis, MG 30/31 was a patient who had two distinct tumors that were present on initial diagnosis. The first (MG 30) was resected and the second tumor (MG31) underwent chemotherapy and radiation followed by resection. To provide further evidence for the latter hypothesis, three previously characterized patient-derived glioma cultures (HK296^24^, HK357^24^, TS603^25^) underwent atac-seq analysis before and after 9 Gray of radiation in three daily fractions. All three cell lines showed a decrease in peak count following radiation (Fig. 4D).

## Discussion

Glioblastoma is a currently incurable disease and despite decades of research and hundreds of clinical trials, the prognosis remains grim. One frustration encountered by several trials^6^ including those investigating the roles of radiation^4^ is that improvements in progression-free survival do not always translate into improvements in overall survival. One possible explanation for this phenomenon is that while certain therapies may initially impede tumor growth those cells that survive may take on additional attributes that make them more destructive and refractory to treatment. The data from this study provides an intriguing new model for this transition. The majority of atac-seq peaks identified in this study were correlated with better survival and tended to positively correlate with genes of nervous system differentiation. There was a non-significant trend for total peak count to decrease as the grade increased with the most significant decrease seen in the recurrent grade IV samples. It may be the case, that the closing of DNA regions responsible for driving neural differentiation induces more malignant behavior and that this process can be accelerated by radiation. If this were true, it may be beneficial to pair radiation therapy with other compounds that could potentially minimize this unwanted side effect.

With advancements in the availability and cost of sequencing, traditional histological classifications have been replaced with more sophisticated genetic markers. Despite the wealth of glioma expression data, attempts to identify patterns of gene expression that predict survival have been underwhelming^32,33^. In contrast, epigenetic data such as DNA methylation (e.g. G-CIMP) seems to have a high degree of prognostic value^18,32,34^. Supporting this observation, this study created an accurate prognostic survival model with only six atac-seq peaks, four of which remained significant when controlling for grade, age and IDH1 status.

The discovery of the IDH1^35^ and H3K27M^36,37^ mutations has revealed the importance of epigenetic dysfunction in the early stages of cancer development. The presumed mechanism of the IDH1 mutation is to favor DNA methylation by inhibiting DNA demethylase enzymes (e.g. Tet family)^18,19^. This is hypothesized to induce decreased DNA accessibility with subsequent gene down-regulation^20^. In the current study, UMAP clustering divided samples by IDH1 status although several IDH1 mutant samples clustered with the IDH1 wildtype group. Running counter to the hypothesis that the IDH1 mutation leads to hyper-methylation and decreased DNA accessibility, there was a trend for IDH1 mutant samples to have more total peaks. This implies that the relationship between DNA methylation and DNA accessibility may be more complicated than previously assumed.

The relative ease and low input requirement of atac-seq technology has led to wide-spread use on a variety of tissue types^38–40^. However, while the data is easy to obtain it is challenging to interpret without additional sources of supporting evidence. In the absence of this supporting evidence several assumptions have become common in the literature namely that a given peak positively regulates the nearest gene. In the current study, the authors assembled a sufficiently large dataset to create a correlation matrix to make some rational assumptions about the relationships of peaks and target genes. Using relatively strict cut-offs, the data showed that most peaks were correlated with one or at most a few genes. The majority of peaks correlated positively with target gene expression. However, there was no correlation between peaks and the nearest genes. On the contrary, peaks and correlated genes were frequently separated by long distances and including many intervening genes. It should be noted that while this study provides some evidence of specific intergenic regions regulating specific target genes, the data is correlational and indirect and needs to be strengthened by alternative methods such as genetic manipulation (CRISPR) or chromosome conformation capture-based methods.

In conclusion, this study uses an innovative peak:gene correlation matrix to create an epigenetic gene regulatory map of gliomas and then uses this matrix to both identify specific gene regulatory regions of interest as well as introduce a possible mechanism of epigenetic malignant progression.

## Data Availability

All sequencing data will be made publicly available.

## References

[1] Stupp, R. *et al.* Radiotherapy plus concomitant and adjuvant temozolomide for glioblastoma. *N. Engl. J. Med.* **352**, 987–996. 10.1056/NEJMoa043330 (2005). 15758009 10.1056/NEJMoa043330

[2] Cancer Genome Atlas Research Network. Comprehensive, integrative genomic analysis of diffuse lower-grade gliomas. *N. Engl. J. Med.* **372**, 2481–2498. 10.1056/NEJMoa1402121 (2015). 26061751 10.1056/NEJMoa1402121 PMC4530011

[3] Bell, E. H. *et al.* Comprehensive genomic analysis in NRG oncology/RTOG 9802: A phase III trial of radiation versus radiation plus procarbazine, lomustine (CCNU), and vincristine in high-risk low-grade glioma. *J. Clin. Oncol.* **38**, 3407–3417. 10.1200/JCO.19.02983 (2020). 32706640 10.1200/JCO.19.02983 PMC7527157

[4] Karim, A. B. *et al.* Randomized trial on the efficacy of radiotherapy for cerebral low-grade glioma in the adult: European Organization for Research and Treatment of Cancer Study 22845 with the Medical Research Council study BRO4: An interim analysis. *Int. J. Radiat. Oncol. Biol. Phys.* **52**, 316–324. 10.1016/s0360-3016(01)02692-x (2002). 11872276 10.1016/s0360-3016(01)02692-x

[5] Baumert, B. G. *et al.* Temozolomide chemotherapy versus radiotherapy in high-risk low-grade glioma (EORTC 22033–26033): A randomised, open-label, phase 3 intergroup study. *Lancet Oncol.* **17**, 1521–1532. 10.1016/S1470-2045(16)30313-8 (2016). 27686946 10.1016/S1470-2045(16)30313-8 PMC5124485

[6] Gilbert, M. R. *et al.* A randomized trial of bevacizumab for newly diagnosed glioblastoma. *N. Engl. J. Med.* **370**, 699–708. 10.1056/NEJMoa1308573 (2014). 24552317 10.1056/NEJMoa1308573 PMC4201043

[7] Brem, H. *et al.* Placebo-controlled trial of safety and efficacy of intraoperative controlled delivery by biodegradable polymers of chemotherapy for recurrent gliomas. The polymer-brain tumor treatment group. *Lancet* **345**, 1008. 10.1016/s0140-6736(95)90755-6 (1995). 7723496 10.1016/s0140-6736(95)90755-6

[8] Johnson, B. E. *et al.* Mutational analysis reveals the origin and therapy-driven evolution of recurrent glioma. *Science* **343**, 189–193. 10.1126/science.1239947 (2014). 24336570 10.1126/science.1239947 PMC3998672

[9] Consortium Group. Glioma through the looking GLASS: Molecular evolution of diffuse gliomas and the Glioma Longitudinal Analysis Consortium. *Neuro Oncol.* **20**, 873–884. 10.1093/neuonc/noy020 (2018). 29432615 10.1093/neuonc/noy020 PMC6280138

[10] Barthel, F. P. *et al.* Longitudinal molecular trajectories of diffuse glioma in adults. *Nature* **576**, 112–120. 10.1038/s41586-019-1775-1 (2019). 31748746 10.1038/s41586-019-1775-1 PMC6897368

[11] Wang, S. I. *et al.* Somatic mutations of PTEN in glioblastoma multiforme. *Cancer Res.* **57**, 4183–4186 (1997). 9331071

[12] Yang, J. M. *et al.* Characterization of PTEN mutations in brain cancer reveals that pten mono-ubiquitination promotes protein stability and nuclear localization. *Oncogene* **36**, 3673–3685. 10.1038/onc.2016.493 (2017). 28263967 10.1038/onc.2016.493 PMC5491373

[13] England, B., Huang, T. & Karsy, M. Current understanding of the role and targeting of tumor suppressor p53 in glioblastoma multiforme. *Tumour Biol.* **34**, 2063–2074. 10.1007/s13277-013-0871-3 (2013). 23737287 10.1007/s13277-013-0871-3

[14] Cairncross, G. & Jenkins, R. Gliomas with 1p/19q codeletion: a.k.a. oligodendroglioma. *Cancer J.* **14**, 352–357. 10.1097/PPO.0b013e31818d8178 (2008). 19060598 10.1097/PPO.0b013e31818d8178

[15] Eckel-Passow, J. E. *et al.* Glioma groups based on 1p/19q, IDH, and TERT promoter mutations in tumors. *N. Engl. J. Med.* **372**, 2499–2508. 10.1056/NEJMoa1407279 (2015). 26061753 10.1056/NEJMoa1407279 PMC4489704

[16] Smith, J. S. *et al.* PTEN mutation, EGFR amplification, and outcome in patients with anaplastic astrocytoma and glioblastoma multiforme. *J. Natl. Cancer Inst.* **93**, 1246–1256. 10.1093/jnci/93.16.1246 (2001). 11504770 10.1093/jnci/93.16.1246

[17] Trent, J. *et al.* Evidence for rearrangement, amplification, and expression of c-myc in a human glioblastoma. *Proc. Natl. Acad. Sci. U.S.A.* **83**, 470–473. 10.1073/pnas.83.2.470 (1986). 3001737 10.1073/pnas.83.2.470 PMC322881

[18] Noushmehr, H. *et al.* Identification of a CpG island methylator phenotype that defines a distinct subgroup of glioma. *Cancer Cell* **17**, 510–522. 10.1016/j.ccr.2010.03.017 (2010). 20399149 10.1016/j.ccr.2010.03.017 PMC2872684

[19] Xu, W. *et al.* Oncometabolite 2-hydroxyglutarate is a competitive inhibitor of alpha-ketoglutarate-dependent dioxygenases. *Cancer Cell* **19**, 17–30. 10.1016/j.ccr.2010.12.014 (2011). 21251613 10.1016/j.ccr.2010.12.014 PMC3229304

[20] Turcan, S. *et al.* IDH1 mutation is sufficient to establish the glioma hypermethylator phenotype. *Nature* **483**, 479–483. 10.1038/nature10866 (2012). 22343889 10.1038/nature10866 PMC3351699

[21] Louis, D. N. *et al.* The 2021 WHO classification of tumors of the central nervous system: A summary. *Neuro Oncol.* **23**, 1231–1251. 10.1093/neuonc/noab106 (2021). 34185076 10.1093/neuonc/noab106 PMC8328013

[22] Habib, N. *et al.* Massively parallel single-nucleus RNA-seq with DroNc-seq. *Nat. Methods* **14**, 955–958. 10.1038/nmeth.4407 (2017). 28846088 10.1038/nmeth.4407 PMC5623139

[23] Buenrostro, J. D., Wu, B., Chang, H. Y. & Greenleaf, W. J. ATAC-seq: A method for assaying chromatin accessibility genome-wide. *Curr. Protoc. Mol. Biol.* **109**, 21–29. 10.1002/0471142727.mb2129s109 (2015). 10.1002/0471142727.mb2129s109 PMC4374986 25559105

[24] Laks, D. R. *et al.* Large-scale assessment of the gliomasphere model system. *Neuro Oncol.* **18**, 1367–1378. 10.1093/neuonc/now045 (2016). 27116978 10.1093/neuonc/now045 PMC5035518

[25] Rohle, D. *et al.* An inhibitor of mutant IDH1 delays growth and promotes differentiation of glioma cells. *Science* **340**, 626–630. 10.1126/science.1236062 (2013). 23558169 10.1126/science.1236062 PMC3985613

[26] Khorasani, M., Shahbazi, S., Hosseinkhan, N. & Mahdian, R. Analysis of differential expression of microRNAs and their target genes in prostate cancer: A bioinformatics study on microarray gene expression data. *Int. J. Mol. Cell Med.* **8**, 103–114. 10.22088/IJMCM.BUMS.8.2.103 (2019). 32215262 10.22088/IJMCM.BUMS.8.2.103 PMC7081080

[27] Saino, M., Maruyama, T., Sekiya, T., Kayama, T. & Murakami, Y. Inhibition of angiogenesis in human glioma cell lines by antisense RNA from the soluble guanylate cyclase genes, GUCY1A3 and GUCY1B3. *Oncol. Rep.* **12**, 47–52 (2004). 15201957

[28] Bajaj, R. *et al.* IMPAD1 and KDELR2 drive invasion and metastasis by enhancing Golgi-mediated secretion. *Oncogene* **39**, 5979–5994. 10.1038/s41388-020-01410-z (2020). 32753652 10.1038/s41388-020-01410-z PMC7539228

[29] Yang, Y. F. *et al.* IMPAD1 functions as mitochondrial electron transport inhibitor that prevents ROS production and promotes lung cancer metastasis through the AMPK-Notch1-HEY1 pathway. *Cancer Lett.* **485**, 27–37. 10.1016/j.canlet.2020.04.025 (2020). 32417395 10.1016/j.canlet.2020.04.025

[30] Choi, C. H. *et al.* Identification of differentially expressed genes according to chemosensitivity in advanced ovarian serous adenocarcinomas: Expression of GRIA2 predicts better survival. *Br. J. Cancer* **107**, 91–99. 10.1038/bjc.2012.217 (2012). 22644307 10.1038/bjc.2012.217 PMC3389416

[31] Ishii, H. *et al.* FEZ1/LZTS1 gene at 8p22 suppresses cancer cell growth and regulates mitosis. *Proc. Natl. Acad. Sci. U.S.A.* **98**, 10374–10379. 10.1073/pnas.181222898 (2001). 11504921 10.1073/pnas.181222898 PMC56968

[32] Lu, J., Cowperthwaite, M. C., Burnett, M. G. & Shpak, M. Molecular predictors of long-term survival in glioblastoma multiforme patients. *PLoS ONE* **11**, e0154313. 10.1371/journal.pone.0154313 (2016). 27124395 10.1371/journal.pone.0154313 PMC4849730

[33] Verhaak, R. G. *et al.* Integrated genomic analysis identifies clinically relevant subtypes of glioblastoma characterized by abnormalities in PDGFRA, IDH1, EGFR, and NF1. *Cancer Cell* **17**, 98–110. 10.1016/j.ccr.2009.12.020 (2010). 20129251 10.1016/j.ccr.2009.12.020 PMC2818769

[34] Ceccarelli, M. *et al.* Molecular profiling reveals biologically discrete subsets and pathways of progression in diffuse glioma. *Cell* **164**, 550–563. 10.1016/j.cell.2015.12.028 (2016). 26824661 10.1016/j.cell.2015.12.028 PMC4754110

[35] Parsons, D. W. *et al.* An integrated genomic analysis of human glioblastoma multiforme. *Science* **321**, 1807–1812. 10.1126/science.1164382 (2008). 18772396 10.1126/science.1164382 PMC2820389

[36] Chung, C. *et al.* Integrated metabolic and epigenomic reprograming by H3K27M mutations in diffuse intrinsic pontine gliomas. *Cancer Cell* **38**, 334–349. 10.1016/j.ccell.2020.07.008 (2020). 32795401 10.1016/j.ccell.2020.07.008 PMC7494613

[37] Nagaraja, S. *et al.* Histone variant and cell context determine H3K27M reprogramming of the enhancer landscape and oncogenic state. *Mol. Cell* **76**, 965–980. 10.1016/j.molcel.2019.08.030 (2019). 31588023 10.1016/j.molcel.2019.08.030 PMC7104854

[38] Corces, M. R. *et al.* Lineage-specific and single-cell chromatin accessibility charts human hematopoiesis and leukemia evolution. *Nat. Genet.* **48**, 1193–1203. 10.1038/ng.3646 (2016). 27526324 10.1038/ng.3646 PMC5042844

[39] Stergachis, A. B. *et al.* Developmental fate and cellular maturity encoded in human regulatory DNA landscapes. *Cell* **154**, 888–903. 10.1016/j.cell.2013.07.020 (2013). 23953118 10.1016/j.cell.2013.07.020 PMC3962256

[40] Corces, M. R. *et al.* The chromatin accessibility landscape of primary human cancers. *Science* **362**, 1898. 10.1126/science.aav1898 (2018). 10.1126/science.aav1898 PMC6408149 30361341

